# Assessment of Mastectomy Skin Flaps for Immediate Implant-Based Breast Reconstruction

**Published:** 2018

**Authors:** M Radu, C Bordea, A Noditi, A Blidaru

**Affiliations:** * “Carol Davila” University of Medicine and Pharmacy, Bucharest, Romania; ** Institute of Oncology Bucharest “Prof. Dr. Al. Trestioreanu”, Department of Surgical Oncology, Bucharest, Romania

**Keywords:** skin flap thickness, immediate breast reconstruction, breast ultrasound, breast MRI, mammography

## Abstract

**Objectives:** The aim of this study is to identify the appropriate preoperative evaluation methods for the quality of the skin flaps and subcutaneous breast layer using different imaging techniques in correlation with the intraoperative findings and also their importance for the outcome in immediate implant-based breast reconstruction.

**Materials and Methods:** Clinical evaluation, mammography, breast ultrasound and breast MRI were used for the assessment of the mastectomy skin flaps which enhanced the selection of the adequate surgical technique for breast reconstruction. The evaluation started with the skin flap measurement of 50 patients with breast cancer, who were candidates for modified radical mastectomy (MRM) in 2014, using the above-mentioned methods, without immediate breast reconstruction. Consequently, 46 nipple-sparing mastectomies (NSM) and 21 skin-sparing mastectomies (SSM) with immediate breast reconstruction with implants were performed between 2014 and 2017 after having such a preoperative subcutaneous tissue thickness evaluation.

**Results:** The intraoperative findings of the MRM group showed a 90% accuracy for the MRI preoperative evaluation, 87% for the ultrasound, 81% for the mammography and 71% for the pinch test. The preoperative measurements for the patients undergoing SSM or NSM were a criterion for choosing the surgical technique for breast reconstruction. The rate of postoperative complications was low.

**Conclusion:** Preoperative clinical measurements, breast ultrasound, breast MRI and mammography can enhance the prediction of the skin flap thickness and thus lead to a low rate of complications and good aesthetic results in implant-based immediate breast reconstruction.

## Introduction

In the last decades, surgical treatment for breast cancer has been represented mainly by conservative treatment, mastectomy being used only for locally advanced cases or for the extensive disease even in early stages (DCIS). The mastectomy rate has been considerably rising lately, especially in young patients. This change is due to frequent genetic testing, to improving breast reconstruction outcome, especially for immediate reconstructions, and to the fear of local recurrence. Even though at first, the oncological safety of these mastectomy techniques was debatable, it has since been demonstrated. A very important issue when choosing and performing these types of mastectomies is represented by the skin flap quality. Mastectomy flap dissection is an essential factor for this surgery because it is supposed to achieve total excision of the breast tissue and preservation of a well-vascularized skin envelope. (**1**) (**2**)

The skin covering the breast has different thicknesses: it is thicker at the periphery of the mammary gland and near the inframammary fold and it is thinner next to the nipple-areola complex. Remnants of the crests of Duret that attach the superficial breast lobes to the superficial layer of the fascia and Cooper’s ligaments were blamed for the inferiority of the oncological safety in skin or nipple sparing mastectomies. In radical mastectomies, the elliptical incision contains the areola and the tumor location and underneath the remaining skin flaps there is as much of the crests of Duret and residual gland as in a subcutaneous mastectomy, so the oncological safety of the skin and nipple sparing mastectomy is accepted in nowadays. (**3**)

This study is looking to identify the appropriate methods for preoperative evaluation of the skin flap thickness using clinical examination, breast ultrasound, breast MRI and mammography, the correlation with the intraoperative findings and also its importance for the aesthetic outcome after immediate implant-based reconstruction. The appropriate surgical method of implant based immediate breast reconstruction should be done knowing that the skin flap thickness can help the surgeon choose the adequate implant placement and avoid complications like skin flap necrosis, implant animation and implant edge visibility.

## Materials and methods

**Fig. 1:**
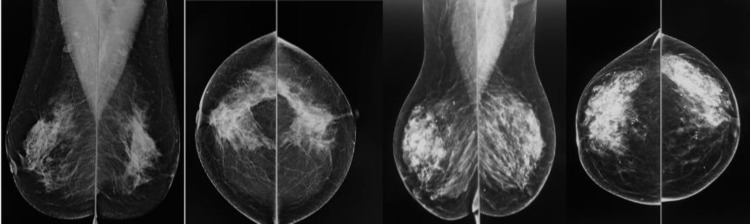
Mammography of two patients with thick subcutaneous flaps. There are two views for each patient: mediolateral oblique view (MLO) and craniocaudal view (CC).

**Fig. 2:**
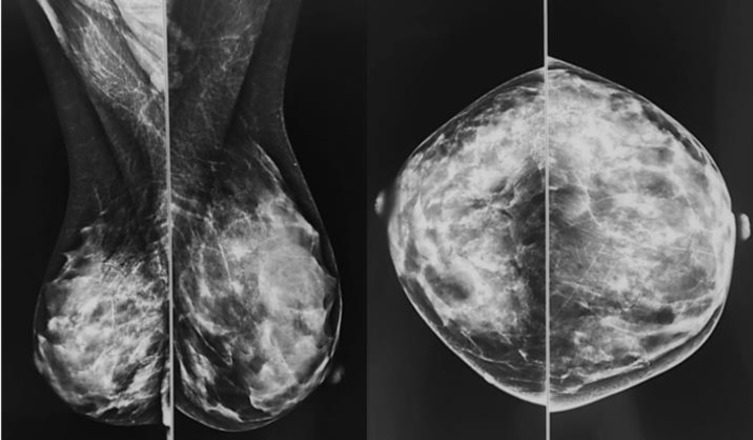
Mammography of a patient with thin subcutaneous flaps in 2 views: mediolateral oblique view (MLO) and craniocaudal view (CC)

**Fig. 3:**
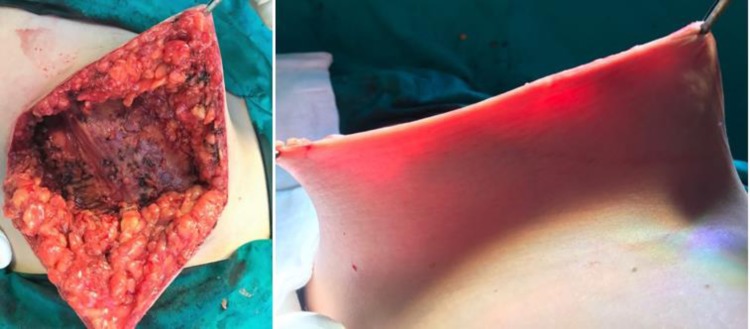
Intraoperative transillumination of the skin flaps after mastectomy - Thick flaps

**Fig. 4:**
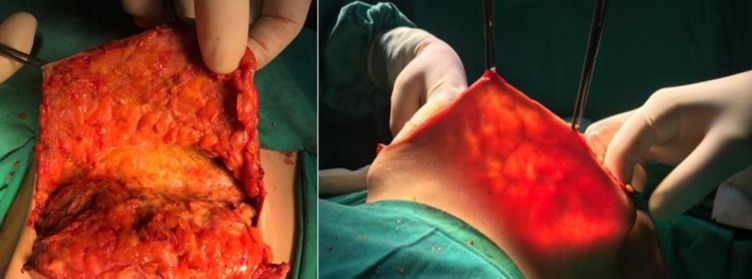
Intraoperative transillumination of the skin flaps after mastectomy - Thin flaps

There is a variability in the subcutaneous breast tissue distinguished on a mammography (**Figure 1**, **2**) that could be correlated with the intraoperative aspect using a simple qualitative method – transillumination (**Figure 3**, **4**), so the purpose of the study is to make a more accurate preoperative evaluation and compare it to the intraoperative one.

The evaluation started with the skin flap measurements, which include the skin and the subcutaneous breast layer using clinical examination, breast ultrasound, breast MRI and mammography on 50 patients with breast cancer, who were candidates for modified radical mastectomy (MRM) in 2014. These patients were not candidates for immediate breast reconstruction. The measurements were taken on the **midclavicular line** to the **nipple,** 5 cm above the nipple, in the middle of the distance between the nipple and the inframammary fold and on the inter-nipple line, at 5 cm medial and lateral to the nipple.

**Fig. 5:**
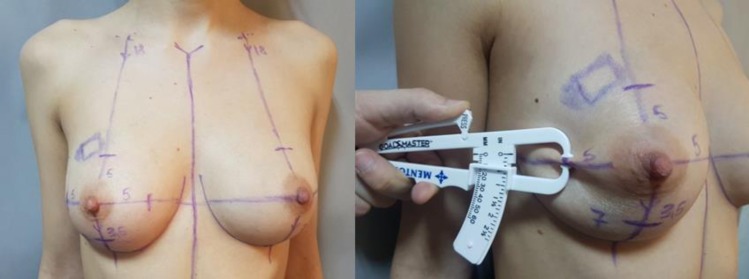
Clinical examination – Pinch test in the established points.

**Fig. 6:**
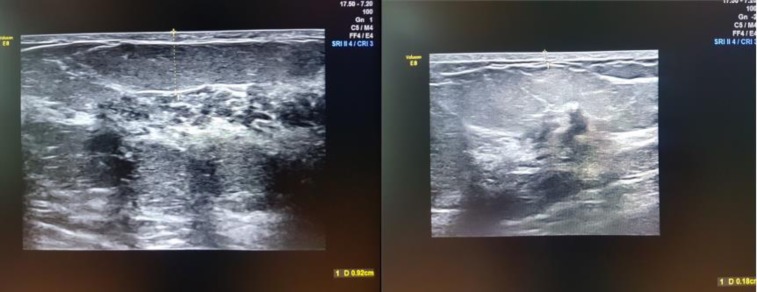
Preoperative breast ultrasound – subcutaneous tissue measurement and skin thickness in the established points.

**Fig. 7:**
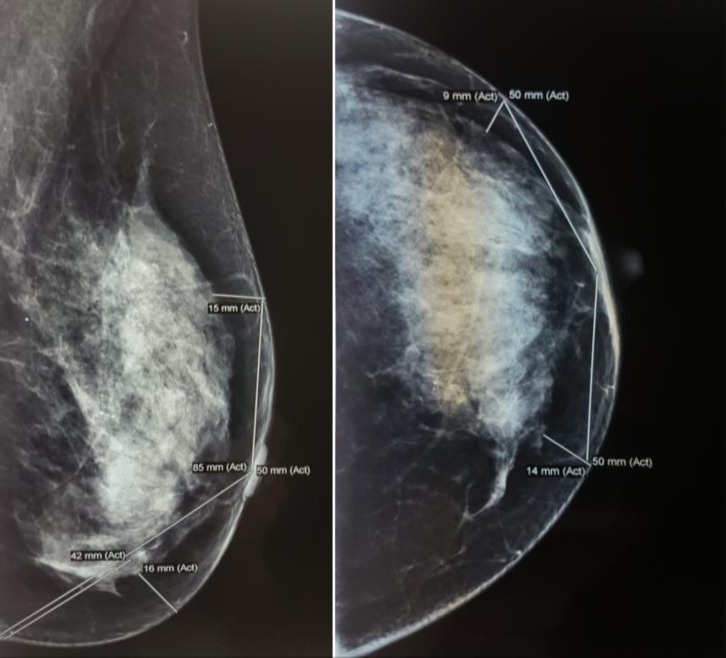
Preoperative left breast mammography. Measurements of the subcutaneous tissue in the established points in 2 views: MLO and CC

**Fig. 8:**
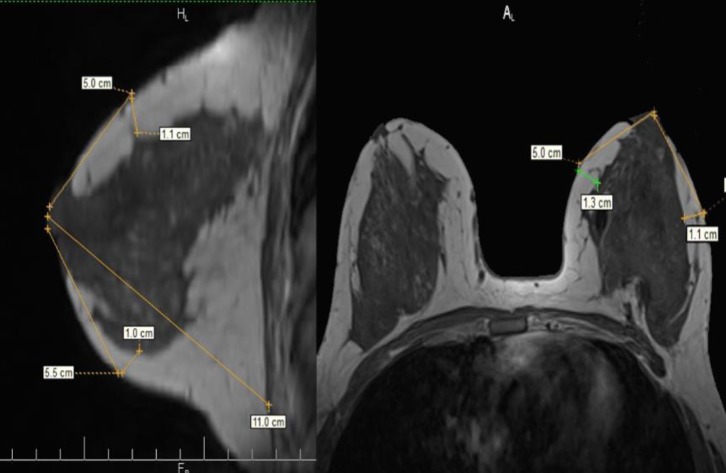
Preoperative breast MRI in T1 sequence without fat saturation. Measurements of the subcutaneous layer in the established points in sagittal and transverse sections.

**Fig. 9:**
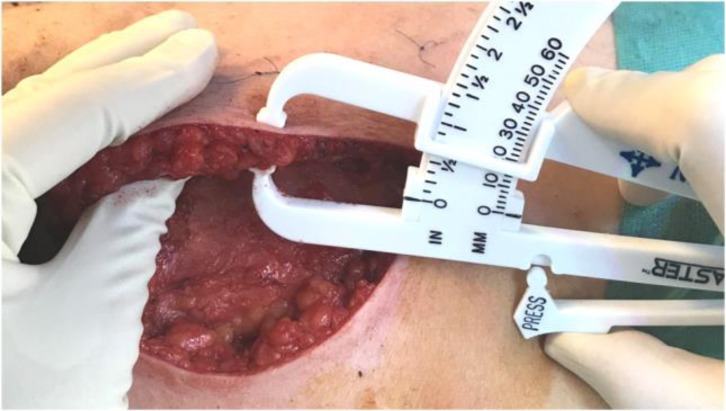
Intraoperative measurement of the skin flaps after mastectomy in the established points.

The clinical examination consisted of the “pinch test” which measures the thickness of the breasts’ skinfolds (**Figure 5**). The measurements during ultrasound used the skin markings that were drawn before the examination in orthostatic position as indicators. The skin thickness alone was also measured separately. (**Figure 6**). On the mammography, the measurements were performed on the mediolateral oblique view (MLO) for the superior and inferior pole and the craniocaudal view (CC) for the medial and lateral part of the breast. (**Figure 7**). Breast MRI was used especially in the T1 sequence without fat saturation where the adipose tissue can easily be seen and the distance between the epidermis and the breast parenchyma was measured at the same levels. (**Figure 8**). Because, during the surgery, the patient is in a dorsal decubitus position, the intraoperative measurement (**Figure 9**) was made in the skin-marked areas determined preoperatively with the patient in the orthostatic position. All the preoperative measurements were made by one surgical team and the intraoperative measurements were made by another surgical team in order to ensure a blind matching.

The next step was to use the preoperative measurements for the 67 immediate implant-based breast reconstructions (IBBR) performed on 60 patients after nipple-sparing (NSM) or skin-sparing mastectomy (SSM), operated between 2014 and 2017 by the same surgical team, at the Institute of Oncology “Prof. Dr. Al. Trestioreanu” and some private hospitals. Unilateral mastectomy with IBBR was performed on 53 patients and 7 patients underwent bilateral mastectomy with IBBR: 2 for bilateral breast cancer and 5 risk-reducing prophylactic mastectomies for unilateral breast cancer and BRCA 1 or 2 mutations. The results of the preoperative measurements were one criterion for choosing the surgical technique for breast reconstruction. Considering the preoperative evaluation of the skin flap thickness, the implants were placed completely under the pectoralis major muscle and anterior serratus (for entire skin flaps thinner than 10mm), partially under the pectoralis and covered by a synthetic mesh in the inferolateral pole (for skin flaps in the upper pole thinner than 10 mm) or above the muscles, covered entirely by the mesh 360° or 180° (for entire skin flaps thicker than 10 mm).

Besides the estimated flap thickness, there were other factors related to the therapeutic decision – oncological factors: stage, the extension of the disease, tumor location, preoperative treatment and the possibility of postoperative radiotherapy; anatomical factors: macromastia, ptosis, BMI, and the personal preference of the patients.

Only the patients with a maximum grade 2 ptosis were included in the study. Patients with macromastia were not included.

The subjective scale used for evaluating the aesthetic result regarding breast shape, breast position, implant visibility and breast animation was: poor, satisfying, good and very good. The evaluation was made 6 months and yearly after the surgery.

## Results

The results, after measuring the skin flaps using clinical examination, breast ultrasound, breast MRI and mammography for the 50 patients with breast cancer, who were candidates for MRM without breast reconstruction, showed: values from 10 mm to 22 mm at the clinical examination, ultrasound 7.8 mm to 15.4mm, mammography 7 mm to 16 mm, MRI 7.9 mm to 13.8 mm. Intraoperative measurements were between 8 mm to 14 mm. The results were compared after the intraoperative evaluation and they showed a 90% accuracy for the MRI preoperative evaluation, 87% for the ultrasound, 81% for the mammography and 71% for the pinch test.

The pinch test revealed the thickest subcutaneous layer, 20% thicker compared to the imagistic methods. The ultrasound, mammography and MRI estimation were closer to the intraoperative findings, in the same breast the highest difference between the four areas was of 12.5%. Breast MRI evaluation is the most accurate compared to the intraoperative findings.

All the measurements revealed that the adipose tissue is not equally distributed in all the quadrants of the breast. The thickest layer is located near the inframammary fold. Comparing all the preoperative measurements, the lower pole had a 32% thicker subcutaneous tissue. The inner quadrants were 3 mm thicker than the outer quadrants.

The retroareolar space is harder to evaluate preoperatively and repeated frozen sections at this level are the most reliable for obtaining an adequate thickness with the right balance between oncological safety and low risk of nipple-areola complex (NAC) necrosis.

The skin thickness also changes along the breast surface and it was estimated separately. Similar to the subcutaneous layer, the ultrasound measurements showed that the skin is thicker in the inferior pole and inner quadrants of the breast, reaching 2.2 mm.

All 67 immediate breast reconstructions were planned accordingly to the preoperative measurements. 20 NSM with immediate reconstruction with implant and unscathed muscles were performed, 26 NSM with implants partially under the pectoralis and covered by a synthetic mesh in the inferolateral pole, 11 SSM with implants partially under the pectoralis and covered by a synthetic mesh in the inferolateral pole and 10 SSM with implants placed completely under the pectoralis major muscle and anterior serratus. There were no unpredicted intraoperative events that caused changes to the preoperative plan for all 67 breast reconstructions.

**Fig. 10:**
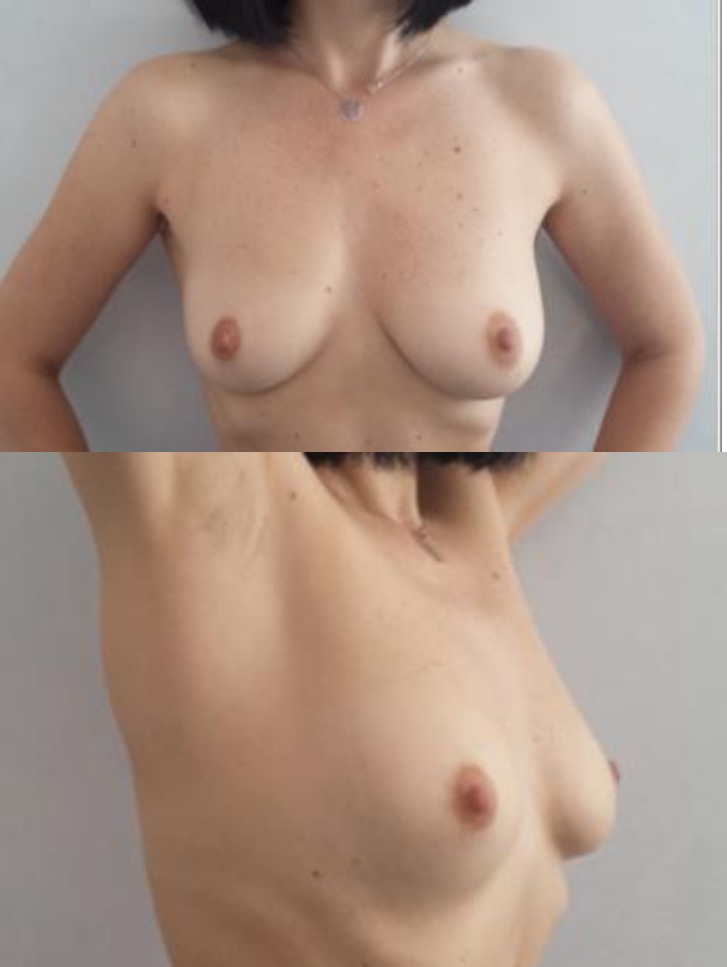
Right breast cancer. Preoperative aspect.

**Fig. 11:**
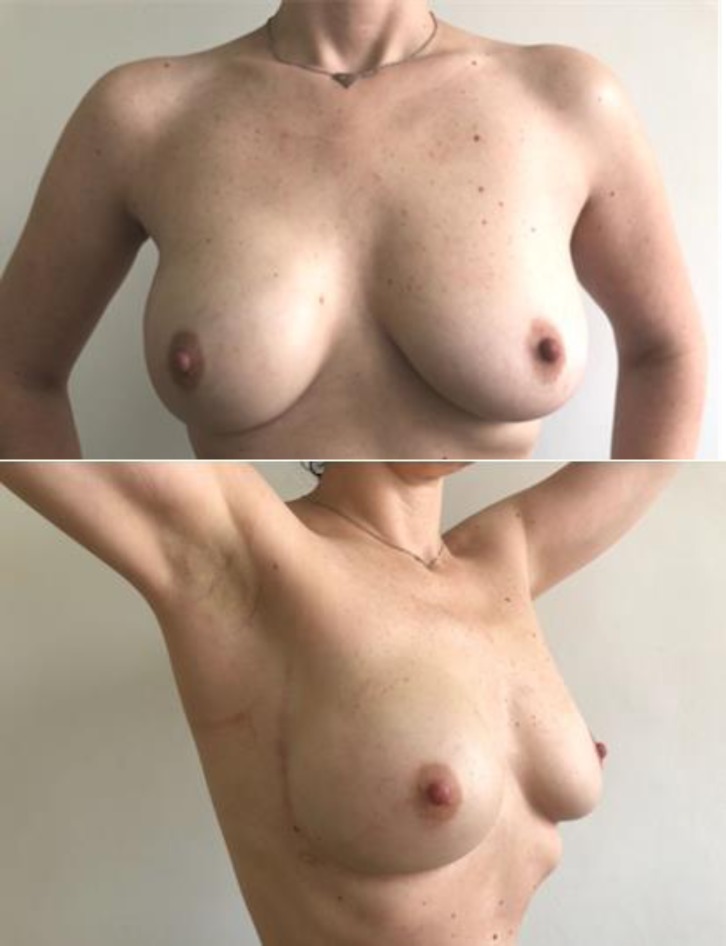
Preoperative skin flap measurement of 15 mm. Right NSM with epipectoral immediate breast reconstruction using an anatomical implant CPG 321, 315 CC and Seragyn mesh. Postoperative aspect after 6 months

Analyzing the aesthetic results of the 46 NSM and 21 SSM for which the preoperative measurements were taken into account when choosing the adequate surgical technique for the breast reconstruction, they were considered very good by both patients and surgical team. (**Figure 10**, **11**).

The complication rate considering skin flap or NAC necrosis was 6%. There was one partial skin flap necrosis after an SSM with a wise-pattern incision and an immediate breast reconstruction using a Becker implant and two partial NAC necroses that were treated conservatively. Having the incision very close to the NAC might have been responsible for the interruption of the nipple vascularization. Only one complete NAC necrosis needed reoperation: skin excision and implant replacement (the anatomical implant was replaced with an Expander).

After a 2-year median follow-up, one local recurrence occurred in a 26-year-old patient with a BRCA1 mutation who refused any adjuvant oncological treatment. The patient had a significant family history – mother diagnosed with breast cancer and a 28-year-old sister who died 2 years after being diagnosed with breast cancer. In this case, the recurrence appeared at 18 months postoperatively.

Lipofilling was not used on any patient. In three patients with BMI under 28 and implants placed above the muscular plane, rippling and wrinkling were noticed. The thickness of the implant coverage for these patients was less than 12mm.

The aesthetic results deteriorated in two patients after receiving postoperative radiotherapy with skin retraction, ascending implants and capsular contracture.

## Discussion

The purpose of the NSM and the SSM is to completely excise the mammary glandular tissue while keeping adequate skin coverage for immediate breast reconstruction. Even in radical mastectomies, it is impossible to excise 100% of the breast parenchyma and the amount of this remaining tissue is directly proportional to the recurrence risk. Careful dissection should be done especially in the axillary tail, chest wall and retroareolar. (**4**)

The superficial breast fascia is the main anatomical plane that should be followed during skin flap dissection, but its existence is controversial and it is present in 56% of patients. A reasonable guide should be the plane between the breast parenchyma and subdermal fat, not necessarily corresponding to the subcutaneous fascial layer. (**5**)

The superficial layer was absent in 44% of the resection specimens and in 42% of the tissues where the superficial layer was present, it contained islands of breast tissue. Beyond the superficial layer, there was no breast tissue identified. A distance of at least 10 mm was encountered in only 5% of the specimens, so even if the superficial fascia was present, because of the small distance to the overlying skin, the dissection superficial to the fascia would not leave viable skin flaps in skin-sparing mastectomies. (**6**)

Oncologic surgeons have long noted a plexus at the subcutaneous level where the breast tissue can be separated from the subcutaneous layer in a relatively avascular plane, and dissection at this plane would allow optimum preservation of the subdermal plexus in order to maintain skin perfusion. (**7**)

The skin flap thickness is a critical factor for the aesthetic outcome and the complication rate in immediate implant-based reconstruction, so its measurement can be used for establishing appropriate candidates for this type of surgery. Surgeons can also be aware of the presence of sections of the breast where the dissection might be difficult because of the breast parenchyma coming too close to the skin envelope. Of course, the mammary gland is replaced with fatty tissue in elderly patients and the breast parenchyma is supposed to be far from the skin, but for young patients, where the breast is very dense, determining the distance to the skin might be of great importance.

There are only a few studies which mention breast ultrasound and mammography as methods for preoperative skin flap evaluation, but this paper has demonstrated that these methods are reliable and simple. Mammography and breast MRI measurements have limitations because of the impossibility to calculate the 5 cm distance from the nipple on the breast surface, having a straight line instead of a curved one. Moreover, for the breast MRI, the patient is in the ventral decubitus position.

Preoperative and postoperative breast magnetic resonance imaging was used in several studies to establish the difference between the flap thickness and its importance relative to the risk of flap necrosis. J.D. Frey et al. showed a difference between the estimated preoperative and postoperative skin-subcutaneous tissue thickness using MRI evaluation. The average total preoperative skin-subcutaneous tissue for nipple-sparing mastectomies (NSM) flap thickness was 11.4 mm. The average total postoperative flap thickness was 8.7 mm. A postoperative flap thickness of less than 8.0 mm was found to be an independent predictor of ischemic complications. (**8**)

The low complication rate in this study was a consequence of implant and surgical technique selection according to preoperative measurements.

When choosing the suprapectoral placement of the implant, the adipose tissue in the upper outer quadrant to the axillary tail and inner quadrants is responsible for not having a visible implant edge. However, if the contour of the implant is visible, the shape of the reconstructed breast can be improved by lipofilling techniques. Clinical evaluation through the use of the pinch test was highly correlated with the aesthetic result and patients with a pinch test of at least 1.2 cm in the superior pole had a more natural aspect of the reconstructed breast. The pinch test can be influenced by the grade of the breast ptosis.

The results of the study with skin thicknesses of up to 2.2 mm are similar to those in the literature. (**9**)

The subcutaneous fat was dependent on the patients’ BMI and age – overweight and older patients have a thicker adipose tissue beneath the skin. For the same reason, mammography is a better predictor for older patients and the ultrasound for the young ones. Breast MRI measuring results were not age-related.

Flap thickness is not the only parameter responsible for the skin or nipple areola necrosis.

The risk factors for skin flap ischemia are controversial. Evaluating breast vascularity with preoperative MRI can be considered when planning an NSM because the presence of dual blood supply to the breast is associated with a decreased risk of nipple-areola complex and skin flap ischemia and necrosis after NSM. After studying 164 NSM where nipple-areola complex or skin flap ischemia or necrosis occurred in 24.4% of cases, there was no association between surgical complications and age, smoking history, BMI, indication for NSM, surgical specimen weight, previous radiation therapy, surgical incision type, reconstruction approach, or operating surgeon on univariate analysis. (**10**)

Unlike previous ones, recent studies have shown that there are predictive patient risk factors for partial or full-thickness necrosis such as smoking, diabetes, obesity, radiotherapy, previous scars and severe medical comorbidity. (**11**)

The incision is also an important risk factor. The risk of skin flaps full thickness necrosis is higher in wise-pattern skin-sparing mastectomy and reaches almost 30%. There were two clusters of hypothetic predictors investigated: patient-related (age, body mass index, smoking, neoadjuvant chemotherapy) and procedure-related (implant weight, breast weight, curative-prophylactic procedure, axillary lymph nodes dissection). Only smoking and weight of prosthesis higher than 468 g showed significant association with skin flap ischemic complications. (**12**)

Another study revealed significant risk factors namely: smoking, young age, type of incision - periareolar and superior circumareolar incisions have a higher risk of necrosis and NAC involvement with areola flap thickness lower than 5 mm. (**13**)

In the present study, patients did not receive radiotherapy prior to surgery. The average BMI was 25.5 and both patients with partial NAC necrosis had a smoking history. The incision for the mastectomy can be crucial for the flap safety. Small incisions are appreciated by the patients but it might need retracting the skin too much and using retractors for an extended period can affect the vascularization of the skin flaps. Most of the NSM in our study were performed using a lateral or inferolateral incision. One incision was radial, close to the NAC and the patient had a partial NAC necrosis.

The flap dissection was made using the electric scalpel. A harmonic scalpel might be a good alternative with decreasing postoperative drainage, seroma development, intraoperative blood loss and wound complications without increasing the operative time. (**14**) Other studies, though, have concluded that it is **not** superior to electrocautery for reducing axillary drainage. (**15**)

Intraoperatively, the viability of the flap tissue can be evaluated using Laser-assisted indocyanine green dye angiography, fluorescein dye angiography or Optical Diffusion Imaging Spectroscopy.

Laser-assisted indocyanine green dye angiography has been compared to fluorescein dye angiography and indocyanine green dye is better for predicting mastectomy skin flap necrosis than fluorescein dye. (**16**) Laser-assisted indocyanine green (ICG) angiography can locate poorly perfused areas intraoperatively. An SPY Elite (®) value of ≤ 7 accurately predicted the development of flap necrosis with 88% sensitivity and 83% specificity. False-positive cases (those with perfusion values ≤ 7 which did not develop necrosis) were more likely to have a smoking history or to have had an epinephrine-containing tumescent solution used during mastectomy. (**17**)

Optical Diffusion Imaging Spectroscopy (ViOptix T.Ox Tissue Oximeter) measures the ratio of oxyhemoglobin to deoxyhemoglobin over a 1 x 1 cm area to obtain a non-invasive measurement of perfusion (StO2). The ability of the ViOptix T.Ox Tissue Oximeter to predict mastectomy flap necrosis was studied and the statistically significant factors contributing to necrosis were: reduction in medial flap StO2, reduction in inferior flap StO2 and flap length. StO2 reductions may be utilized to identify impaired perfusion in mastectomy skin flaps. (**18**)

If skin flap necrosis appears, there are different management options.

Conservative treatment was applied to the 2 patients with partial NAC necrosis. Local wound care: daily changing dressings and debridements along with topical heparin and loose dressings to prevent additional ischemia by compression.

Other substances like Dextran-40 and topical nitroglycerin can be used for preventing or treating necrosis. Dextran-40 treatment did not affect the development of flap necrosis. However, if necrosis had already developed, the necrotic area of the skin flaps improved with dextran-40 treatment. (**19**) Early intervention reduces the morbidity of mastectomy skin flap necrosis (MSFN) in selected cases. Topical nitroglycerin ointment may be beneficial in reducing MSFN following immediate reconstruction, but the evidence base is still limited. (**11**)

For the patient with complete NAC necrosis that needed reoperation with implant replacement, the progression of the skin flap necrosis was observed during the period in which the patient refused the second surgery and was treated conservatively. Excision of the affected skin and the exposed part of the synthetic mesh was performed along with the implant replacement – an Expander was placed instead of the anatomical one.

Tissue necrosis in implant-based immediate reconstructions needs to be treated with more caution and, arguably, more aggressively than autologous tissue reconstructions. (**20**) However, for larger full-thickness wounds, it may be more appropriate to bring the patient back to the operating room soon after mastectomy skin necrosis is observed for debridement, primary closure, and/or skin grafting. Other authors have described a more conservative approach for large areas of skin necrosis considering that early operative intervention creates contour and volume abnormalities that are later more difficult to correct. Healing by secondary intention also eliminates the “patchwork” appearance that skin grafts may produce. Maintaining a moist environment at the borderline skin, applying an antibiotic ointment that penetrates the eschar and promotes separation, and wet to dry dressings over granulating surfaces are effective strategies for this wound care. (**21**)

For a good result after mastectomy and IBBR from both an oncologic and an aesthetic point of view, preoperative measurements are required, such as careful dissection resulting in viable skin flaps and suitable selection of the implant type and placement (pre/retropectoral).

## Conclusion

Preoperative evaluation of the mastectomy skin flap thickness after clinical evaluation, breast ultrasound, breast MRI and mammography can be used for estimating the possibility of immediate implant-based breast reconstruction, the appropriate prosthesis and the surgical procedure. There is a strong correlation between the preoperative measurements and the intraoperative findings and the association of all four methods leads to the best result.
